# Ultrasound-guided microwave ablation for giant breast leiomyoma: A case report

**DOI:** 10.3389/fonc.2023.1095891

**Published:** 2023-01-19

**Authors:** Siqi Zhang, Lu Wang, Jianquan Yang, Man Lu

**Affiliations:** Ultrasound Medical Center, Sichuan Cancer Hospital and Research Institute, Sichuan Cancer Center, School of Medicine, University of Electronic Science and Technology of China, Chengdu, Sichuan, China

**Keywords:** ultrasound_1_, breast_2_, leiomyoma_3_, microwave ablation_4_, therapy_5_

## Abstract

**Rationale:**

Breast leiomyoma is the rarest non-epithelial tumor of the breast. As a benign tumor, its main treatment is regular follow-up. Surgical treatment is often used in clinical practice when patients have symptoms or strongly require treatment. However, if the tumor is large or located around the nipple or areola, the cosmetic effect of surgery is not good, so it is urgent to find new treatment methods. We pioneered the use of microwave ablation in the treatment of giant breast leiomyoma and achieved good results.

**Patient concerns:**

A 37-year-old female was admitted to hospital because she found a breast mass of approximately 8 cm. She had no obvious clinical symptoms, but had great psychological pressure.

**Diagnosis:**

Pathological biopsy showed leiomyoma followingly the surgical operation of giant breast leiomyoma was planned. However, the breast mass was large, and the postoperative scar would affect the breast appearance.

**Interventions:**

The consent was obtained from the patient and her family. The Ultrasound-guided microwave ablation was successfully performed.

**Outcomes:**

The patient was followed up for 10 months, and the tumor volume ablation rate was 69.8%. The cosmetic effect of breast was excellent.

**Lessons:**

To our knowledge, this is the first case to using microwave ablation (MWA) for the treatment of breast leiomyoma. Ultrasound-guided MWA can be used for the treatment of breast leiomyoma, especially when the mass is large and requires traditional surgical resection. It can effectively improve the breast aesthetics and further improve the quality of life of patients. However, it is only a case report, and needs more research to verify MWA in breast leiomyoma.

## 1 Introduction

Breast leiomyoma is considered as the rarest non-epithelial tumor of the breast, accounting for less than 1% of all breast neoplasms (1, 2), and it usually occurs in middle-aged women. At present, the clinical manifestations, imaging features and differential diagnosis of breast leiomyoma have been reported in the related studies (3–5), but the treatment of breast leiomyoma is rare. In clinical practice, traditional surgical resection is often chosen for the treatment of large breast leiomyomas. However, postoperative scars can affect the appearance of the breast, or cause nipple traction and tilt or damage to the breast ducts. It’s urgent to find new treatments to improve the prognosis of patients with large breast masses. We reported a case of giant breast leiomyoma treated by ultrasound-guided microwave ablation (US-MWA) and followed up for 10 months.

## 2 Case report

**Figure 1:**
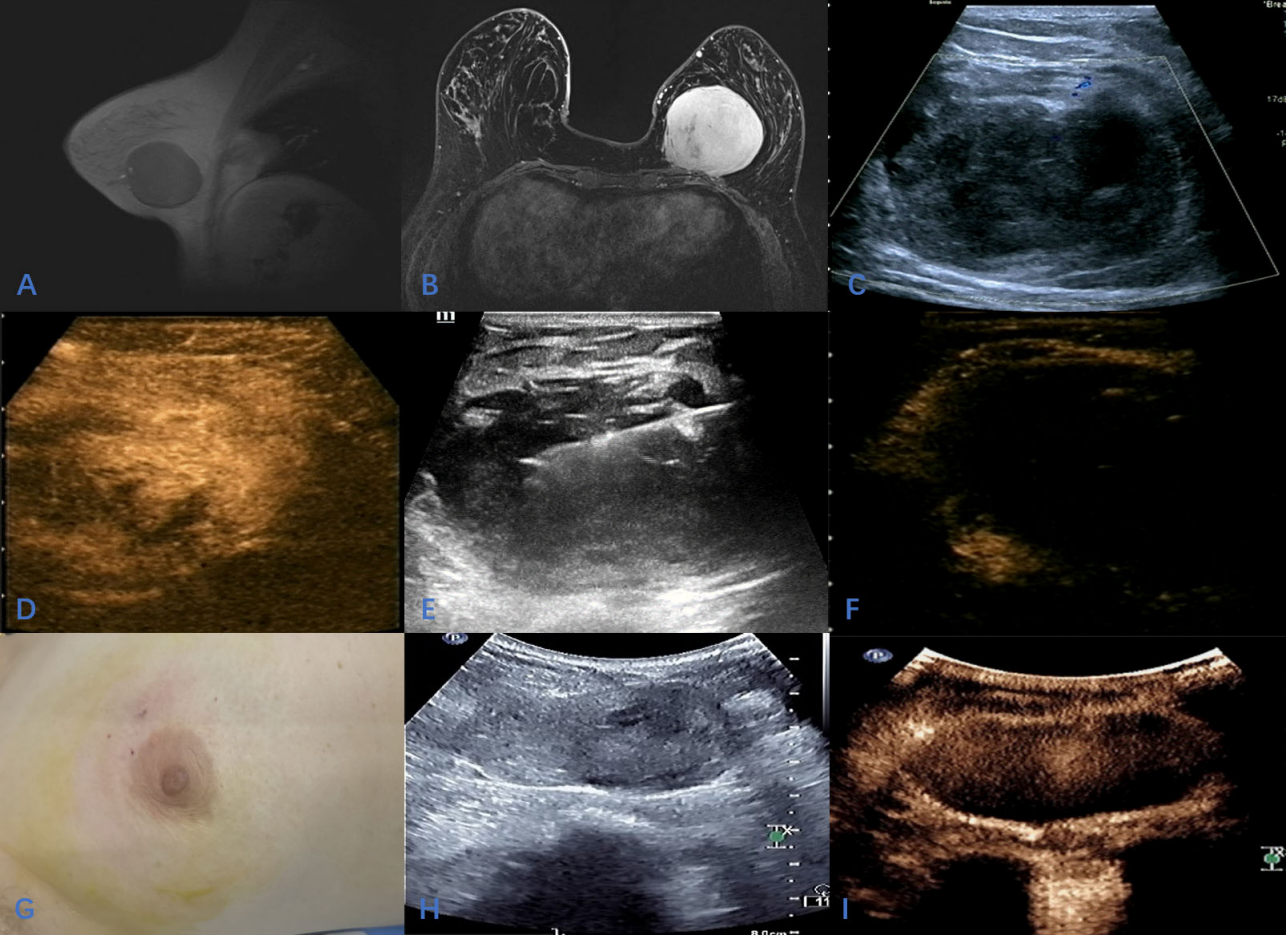
A 37-year-old female with huge mass in the left breast; **(A, B)** The location of the tumor on enhanced MRI; **(C)**; Sonographic appearance of the tumor on 2D-Ultrasound; **(D)** Contrast-enhanced ultrasound showed homogeneous hyperenhancement of the tumor during the arterial phase; **(E)** During ultrasound-guided microwave ablation; **(F)** The area of ablation showed no enhancement in arterial phase and venous phase after ablation; **(G)**; The skin of the breast was slightly ecchymosis after ablation, and the appearance of the breast was intact; **(H, I)** After 10 months of follow-up, two-dimensional ultrasound showed that the mass was significantly reduced, and CEUS showed that there was no enhancement in the arterial phase and venous phase of the ablation area.

**Figure 2:**
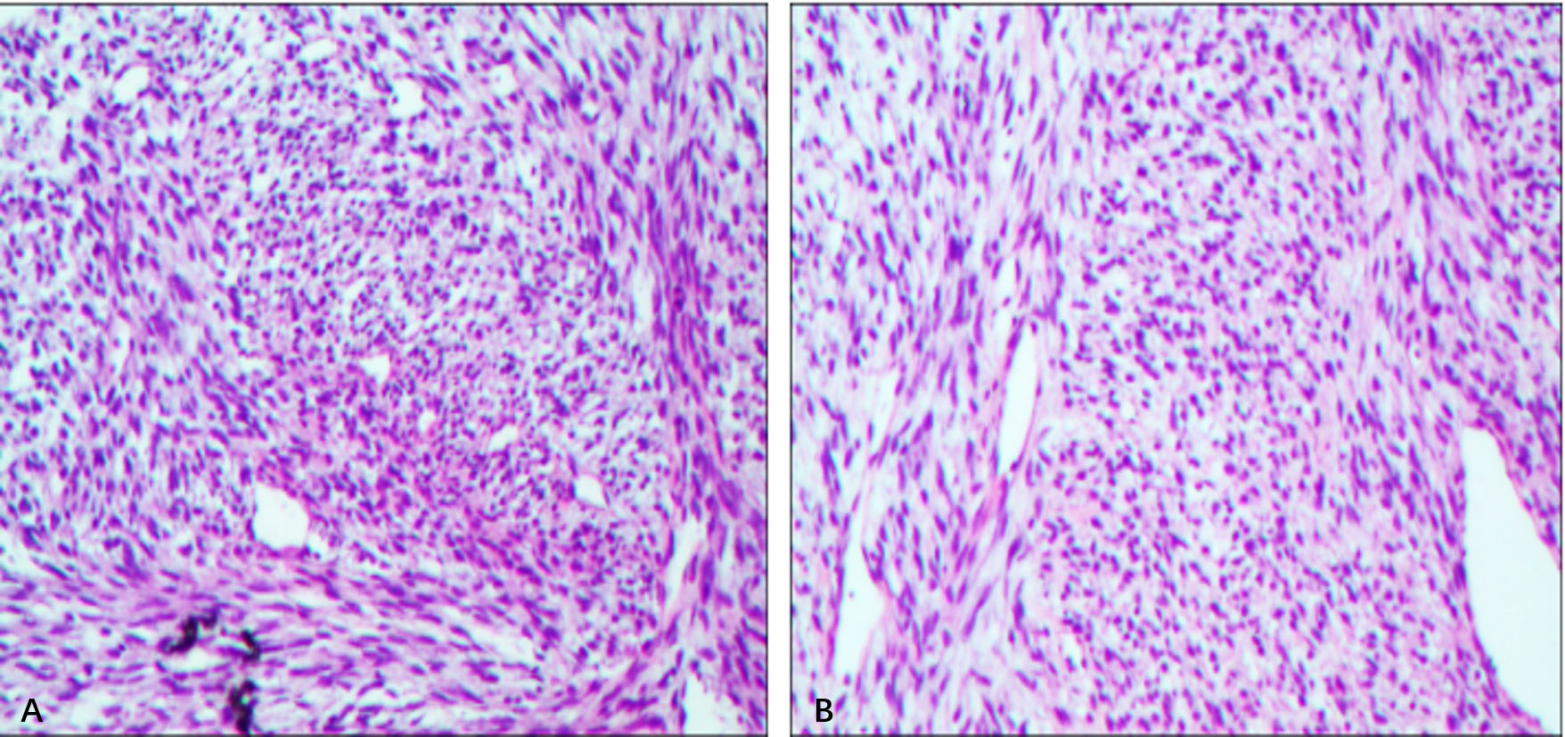
Histological sections revealing monotonous spindle cell proliferation, necrosis or mitotic figures found. **(A, B)** Histologic image of leiomyoma. (H&E, × 400).

A 37-year-old woman complained with a mass in the left breast and visited our hospital. One month ago, the patient accidentally found a goose-egg-sized mass in the lower inner quadrant of the left breast, which was hard but removable. The patient did not have any clinical symptoms, no breast skin change or ipsilateral axillary lymph node enlargement, etc. The patient had been in good health and had no family history of breast cancer. In the inner and lower quadrant gland of the left breast, enhanced MRI revealed a hypoechoic oval mass measuring 7.6*6.6*7.5cm mass that showed progressive enhancement in the arterial phase and diffuse enhancement in the delayed phase. The dynamic enhancement curve (TIC type) of this mass was characterized as plateau type (type II). The mass was classified as being in Breast Imaging-Reporting and Data System (BI-RADS) category 4. It had morphological rules and circumscribed margins, and was coincident with the location described through US (**Figures 1A, B**). The patient was diagnosed as breast leiomyoma by pathological examination *via* sonography guide core needle biopsy (**Figure 2**). Because of the large size of the tumor, the surgeon recommended that the patient undergo traditional surgical resection, but the incision should be at least 4cm. The patient is a young woman who has a strong desire to keep a good-looking appearance of the left breast. Finally, the patient chose to undergo US-MWA to treat the giant leiomyoma of the left breast.

### 2.1 Preoperative examination

No abnormality was found in blood routine, coagulation and pre transfusion examinations. The 2D-Ultrasound Contrast-enhanced ultrasound (CEUS) was performed using Mindry Resona 7 ultrasound system to discover the optimal ablation puncture path (**Figures 1C, D**).

### 2.2 Ablation process

The surgeon was a radiologist with more than 10 years of experience in interventional therapy. Instruments used during the operation include Mindry Resona 7 ultrasound system, microwave ablation instrument (Nanjing Kangyou, 2000KY), sterile disposable microwave ablation needle (3mm in diameter). The operative region was disinfected and shopped towels routinely. The operative procedure was started under local anesthesia. The use of refrigerated saline (3-5°C) to isolate and protect the breast tissue and pectoralis muscle around the tumor before ablation could reduce the high temperature caused by thermal ablation and avoided thermal damage to the surrounding tissues during ablation. The intraoperative ablation power was 20-40W. During the operation, short time, long time interval and multiple ablations were performed. When the hyperechoic area was completely covered by ablation, CEUS examination was performed, and there was no enhancement in the ablation area, indicating that the ablation was completed. (**Figures 1E, F**). Patients were instructed to apply ice at an interval of 24 hours after ablation.

### 2.3 Postoperative follow-up

The patient had postoperative pain in the operation area, and the VAS score was 4. After cold compress, the swelling and pain in the operation area decreased significantly after about 3 days, and returned to normal in about 3 weeks. There was slight ecchymosis, redness and swelling on the skin of the operation area after operation (**Figure 1G**), which disappeared completely after 4 weeks. Patients were reexamined after 10 months of follow-up using Philips EPIQ 7 ultrasound system. CEUS showed no enhancement in the ablation area of the left breast, and the size of the ablation area was 6.0*3.5*5.4cm. The ablation area was completely ablated without recurrence, and the lesion was significantly smaller than before (**Figures 1H, I**). The tumor volume ablation rate was 69.8%.

## 3 Discussion

Breast leiomyoma is a rare benign mesenchymal tumor that presents as an isolated, slow-growing tumor with features similar to those of the most common benign tumors (3, 6). At present, the cell origin of breast parenchymal leiomyoma is not fully understood (7). Several hypotheses have been proposed, including theories of embryonic transposition of smooth muscle cells from smooth muscle hemangioma cells or pluripotent mesenchymal cells and smooth muscle cells in the areola (8–11).

Treatment of breast leiomyomas is controversial. One study reported that the patient had no malignant transformation during a 9-year follow-up period (12). Breast leiomyoma without obvious clinical symptoms can also be similar to other benign breast tumors and regularly followed up. However, it has been reported that in order to avoid local recurrence, the standard recommended treatment is local resection with free margins (13). The patient in this case report underwent microwave ablation for minimally invasive treatment due to high pressure on breast appearance and concerns about excessive tumor volume.

It was our first attempt to ablate such a large breast mass, which was close to the pectoralis major muscle. During the operation, we adopted some ablation technology reforms to reduce the patients’ pain during the operation and improve postoperative prognosis. In this report, the limited experience with ultrasound-guided giant breast leiomyomas is summarized as follows: Firstly, we used refrigerated sterile saline to isolate the tumor from the surrounding tissue, which neutralized the heat generated by thermal ablation, reduced the patient’s pain, and increased the operator’s visibility of the surgical area. Secondly, short time ablation, long time interval and multiple ablations during the operation could maximize the protection of surrounding tissues and adjacent muscle tissues and avoid thermal damage.

To the best of our knowledge, this is the first treatment of breast leiomyoma using MWA. Moreover, this was a case report, and the follow-up period was short and only 10 months. Additional follow-up MRI should have been performed, but the patient declined to undergo MRI because of mild claustrophobia after the initial preoperative MRI, and we used contrast-enhanced ultrasonography for postoperative follow-up. The application of MWA in the treatment of breast leiomyoma still needs multi-center and large sample studies to confirm its efficacy and safety. Patients after ablation should be followed up for a longer period of time to observe whether the absorption pattern of the ablation area after ablation is similar to other benign breast tumors.

## 4 Conclusion

Ultrasound-guided MWA may be used for breast leiomyoma which can completely ablate tumor and retains the integrity of the breast shape, especially when the tumor volume is large, minimally invasive surgery cannot be performed. However, it is only a case report, and needs more research to verify MWA in breast leiomyoma.

## Data availability statement

The original contributions presented in the study are included in the article/supplementary material. Further inquiries can be directed to the corresponding author.

## Ethics statement

The studies involving human participants were reviewed and approved by Cancer Hospital affiliate to School of Medicine, University of Electronic Science and Technology of China (UESTC) Medical Research Ethics Committee. The patients/participants provided their written informed consent to participate in this study.

## Author contributions

All authors listed have made a substantial, direct, and intellectual contribution to the work and approved it for publication. SZ, LW, ML designed the study and drafted the manuscript. SZ, LW, and JY performed data collection and data analysis. SZ wrote the manuscript, and ML supervised the study and critically revised the manuscript. All authors contributed to the article and approved the submitted version.
