# Supplementary material for: Ultrasound-guided microwave ablation for giant breast leiomyoma: A case report
Source: Front Oncol. 2023 Jan 19;13:1095891. doi: 10.3389/fonc.2023.1095891 (PMC9893409; doi:10.3389/fonc.2023.1095891)

First visit

Contrast-enhanced ultrasound and needle biopsy were performed

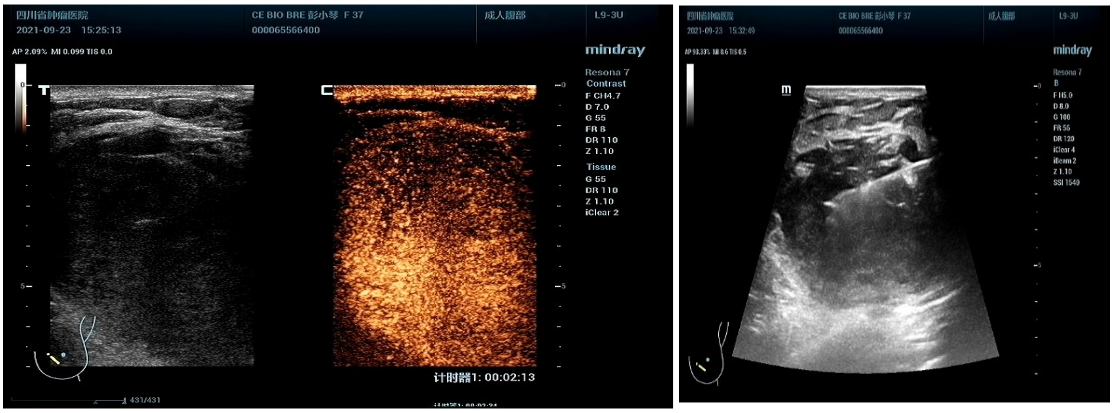

MRI

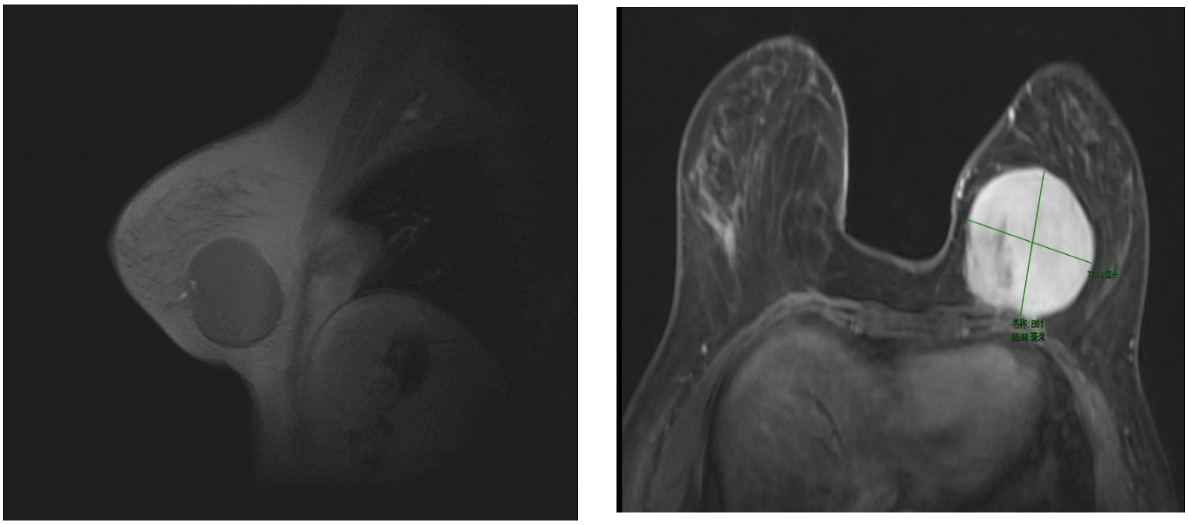

Pathology

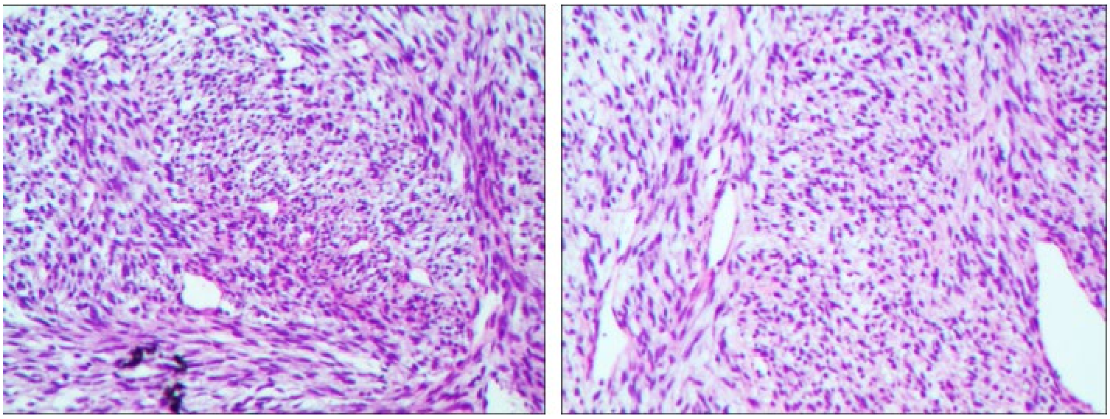

microwave ablation

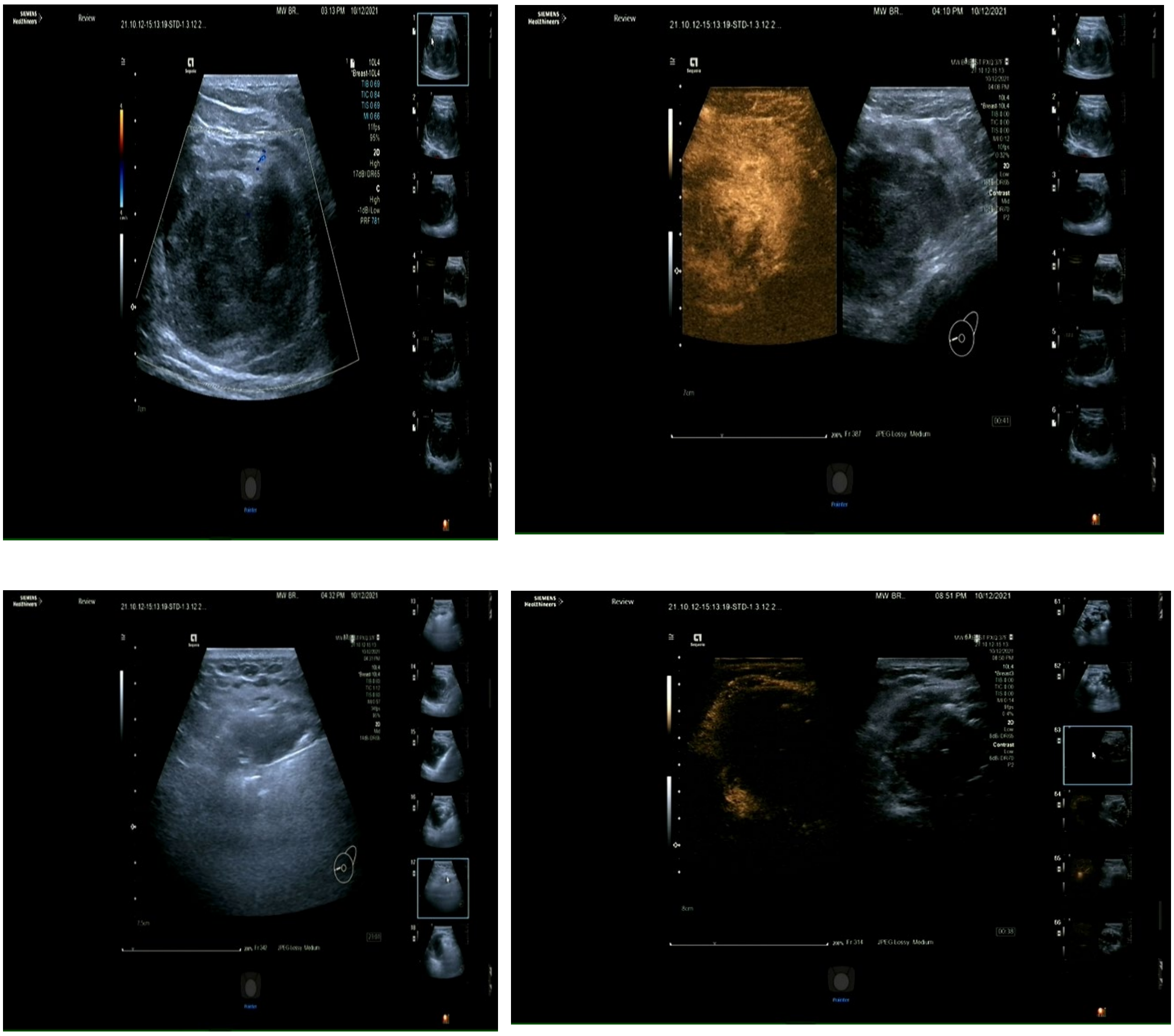

Postoperative breast appearance

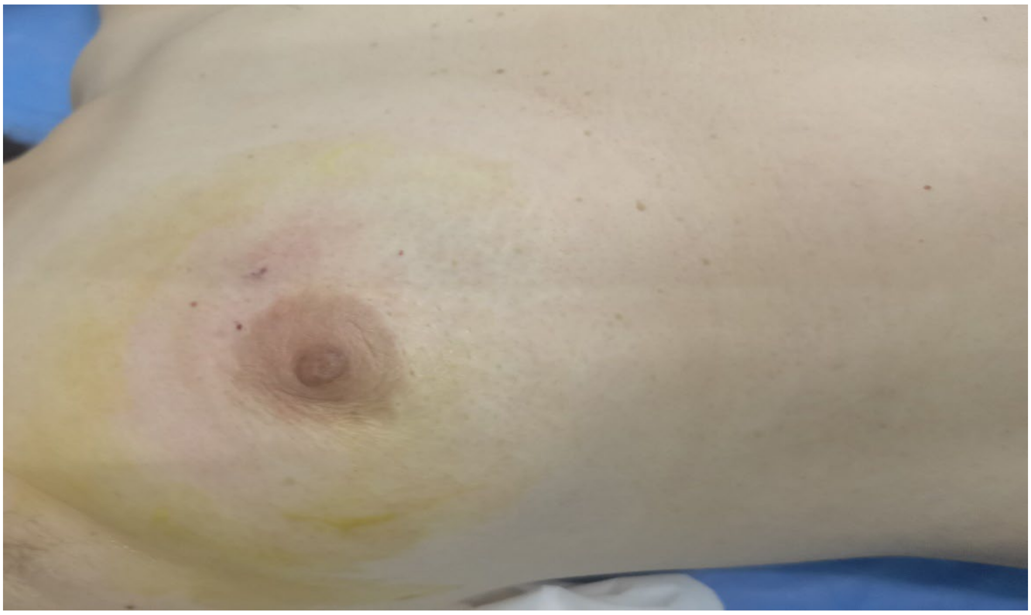

The patients were followed up after 3 months

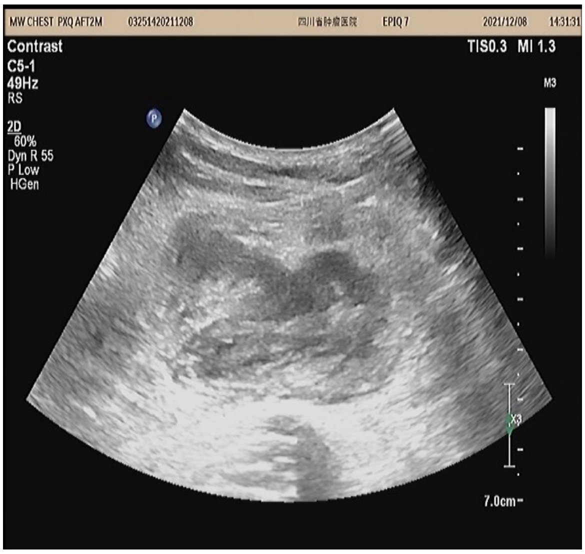

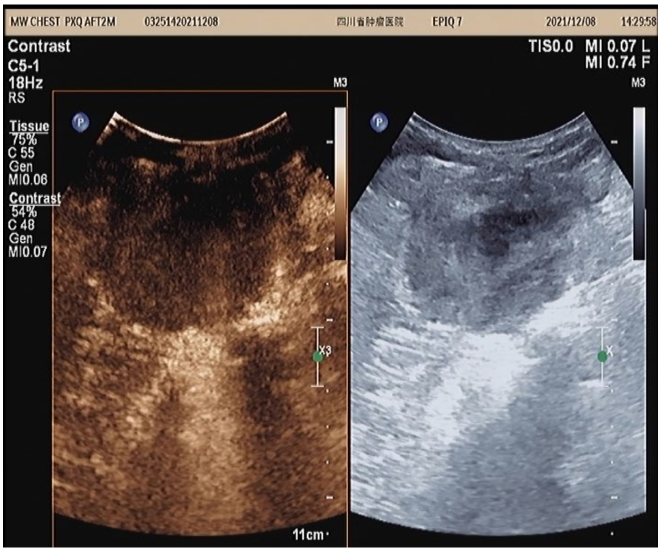

The patients were followed up after 10 months

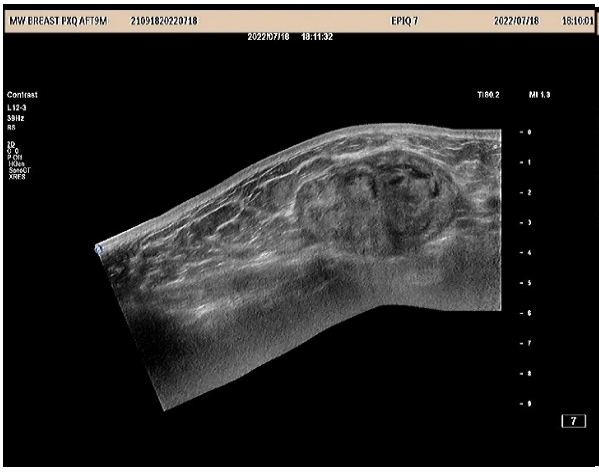

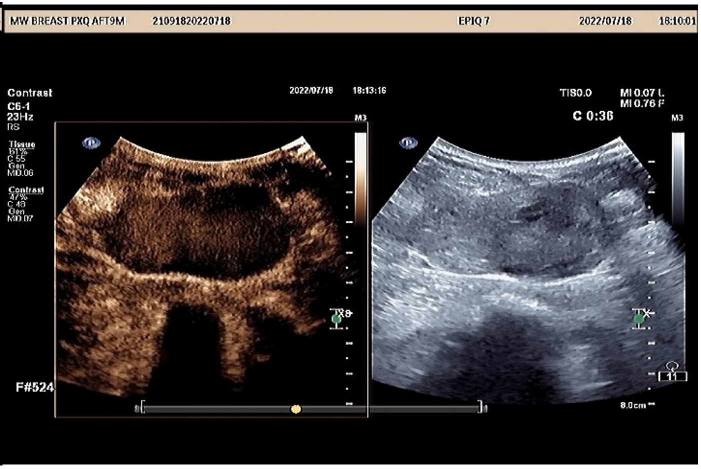

Supplement: Supplementary file 1 [file Image_1.pdf]
